# Advancing recombinant protein production in CHO cells through metabolic engineering

**DOI:** 10.3389/fbioe.2026.1847073

**Published:** 2026-08-03

**Authors:** Lu Hou, Weidong Li, Ziyan Li, Shaolei Geng, Tianyun Wang, Junhe Zhang

**Affiliations:** 1 Institutes of Health Central Plains, Xinxiang Key Laboratory for Tumor Drug Screening and Targeted Therapy, Henan Medical University, Xinxiang, China; 2 International Joint Research Laboratory for Recombinant Pharmaceutical Protein Expression System of Henan, Henan Medical University, Xinxiang, China; 3 Henan Key Laboratory of Neurorestoratology and Protein Modification, The First Affiliated Hospital of Henan Medical University, Xinxiang, China

**Keywords:** biopharmaceutical, Chinese hamster ovary cells, mammalian cell culture, metabolic engineering, recombinant protein production

## Abstract

Chinese hamster ovary (CHO) cells serve as the predominant platform for producing recombinant therapeutic proteins in biopharmaceutical manufacturing, the production capacity of which relies heavily on efficient protein synthesis, folding, and secretion pathways. However, during high-density and prolonged cultivation, these cells frequently encounter bottlenecks—including excessive lactate and ammonia accumulation, redox imbalance, and endoplasmic reticulum (ER) stress—which ultimately constrain both the yield and quality of target protein. To overcome these limitations, metabolic engineering has emerged as a key strategy; through systematic modification of the CHO cellular metabolic network, it enhances recombinant protein yield, optimizes critical product qualities such as glycosylation, and improves overall process robustness. This review summarizes recent advances in CHO cell metabolic engineering, encompassing the regulation of central metabolic pathways, glycosylation engineering, cell cycle and metabolic reprogramming, culture condition optimization, byproduct accumulation control, and the application of systems biology and artificial intelligence technologies, including genome-scale metabolic modeling, machine learning-guided target prediction, and dynamic process control. These advances have significantly reduced biopharmaceutical production costs, improved scalability, and shortened time-to-market for monoclonal antibodies and other complex biologics. As the field transitions from single-gene manipulation toward multi-target, dynamic, and system-level rational design, metabolic engineering is advancing CHO cells into more efficient and intelligent “cell factories”, thereby providing sustained momentum for the industrial production of biologics.

## 1 Introduction

Chinese hamster ovary (CHO) cells serve as a core mammalian expression host, occupying an irreplaceable position in the industrial production of complex biologics, including recombinant therapeutic proteins and monoclonal antibodies (Fischer et al., 2015; Sharker and Rahman, 2021). Their capacity for post-translational modifications closely mirrors that of human cells, facilitating the correct folding and assembly of complex, functional proteins. This makes CHO cells the preferred platform for manufacturing high-safety and high-efficacy biopharmaceuticals (Kim et al., 2012).

In industrial CHO cell applications, metabolic engineering plays an increasingly vital role. Its primary goal is to enhance overall cell factory performance through directed modification of intracellular metabolic networks, optimizing fluxes of carbon and nitrogen while alleviating metabolic bottlenecks (Kim et al., 2012; Kim et al., 2023). These improvements manifest concretely as increased protein titer and specific productivity (qP), better critical quality attributes (e.g., glycosylation homogeneity), enhanced metabolic robustness (defined as the ability to maintain stable growth, productivity, and quality despite environmental fluctuations) during culture, and reduced accumulation of detrimental byproducts such as lactate and ammonia (Toussaint et al., 2016; Wang and Betenbaugh, 2018).

Historically, metabolic engineering relied mainly on overexpression, knockdown, or knockout of single key enzymes, such as lactate dehydrogenase (LDH) and pyruvate dehydrogenase (PDH), which catalyze rate-limiting steps in central carbon metabolism and directly control lactate production (Miao et al., 2018; Wang et al., 2018). In recent years, however, the integration of systems and synthetic biology tools (Choi et al., 2019) has driven a paradigm shift in the field (Kildegaard et al., 2013; Kuo et al., 2018; Tejwani et al., 2018). The focus has moved from single-gene manipulation toward system-level design and dynamic, precise control of metabolic networks (Kavoni et al., 2025). This includes using transcription factor engineering to coordinate pathway expression (Gutierrez-Gonzalez et al., 2019), constructing metabolite-sensing feedback circuits for adaptive regulation, and rationally designing complex traits like glycosylation via multi-gene editing and pathway reconstruction (Routier et al., 1997; Slade et al., 2016). Furthermore, the emergence of “systems metabolic engineering”—which combines genome-scale metabolic models (Rejc et al., 2017), multi-omics data, and artificial intelligence predictions (Fouladiha et al., 2021)—has greatly improved the predictability of target identification and intervention strategies, accelerating the transition from experimental trial-and-error to rational design (Table 1).

**TABLE 1:** Key research domains and engineering strategies for CHO cell metabolic modification.

Research directions	Main strategies	Key technologies	Application effect
Central metabolism engineering	Gene overexpression, Knockdown, Dynamic regulation, Transcription actor engineering	CRISPR/Cas9, Metabolite sensors, Transcription factors	Improve energy efficiency, Reduce lactate accumulation, optimize cofactor balance
Glycosylation engineering	Gene editing, Multi-gene editing, Precursor optimization	CRISPR, Base editors, CHOGlycoNET model	Regulate glycoforms, improve glycoform consistency and ability
Cell cycle regulation	G1 arrest induction, Checkpoint intervention, Apoptosis pathway regulation	CDK4/6 inhibitors, MAT2A silencing, BAX knockout	Increase specific productivity (qP), extend culture duration, enhance metabolic adaptability
Culture condition optimization	Dynamic nutrient feeding, Physicochemical parameter control, Alternative substrate use	Online monitoring, Feedback control, Alternative carbon sources	Maintain metabolic homeostasis, reduce byproduct accumulation, improve product quality and yield
Byproduct regulation	Metabolic pathway remodeling for lactate and ammonium, Small molecule inhibitors, Process optimization	LDH Downregulation, GS pathway enhancement, 2-DG, DCA	Reduce lactate and ammonia accumulation, improve cell growth environment, enhance glycosylation quality
Systems biology and AI	Multi-omics integration, Genome-scale metabolic models, Machine learning prediction, Real-time metabolic monitoring	^13^C-MFA, scRNA-seq, Deep learning models, Raman spectroscopy	Target identification, phenotype prediction, adaptive process control, accelerate rational design

**FIGURE 1:**
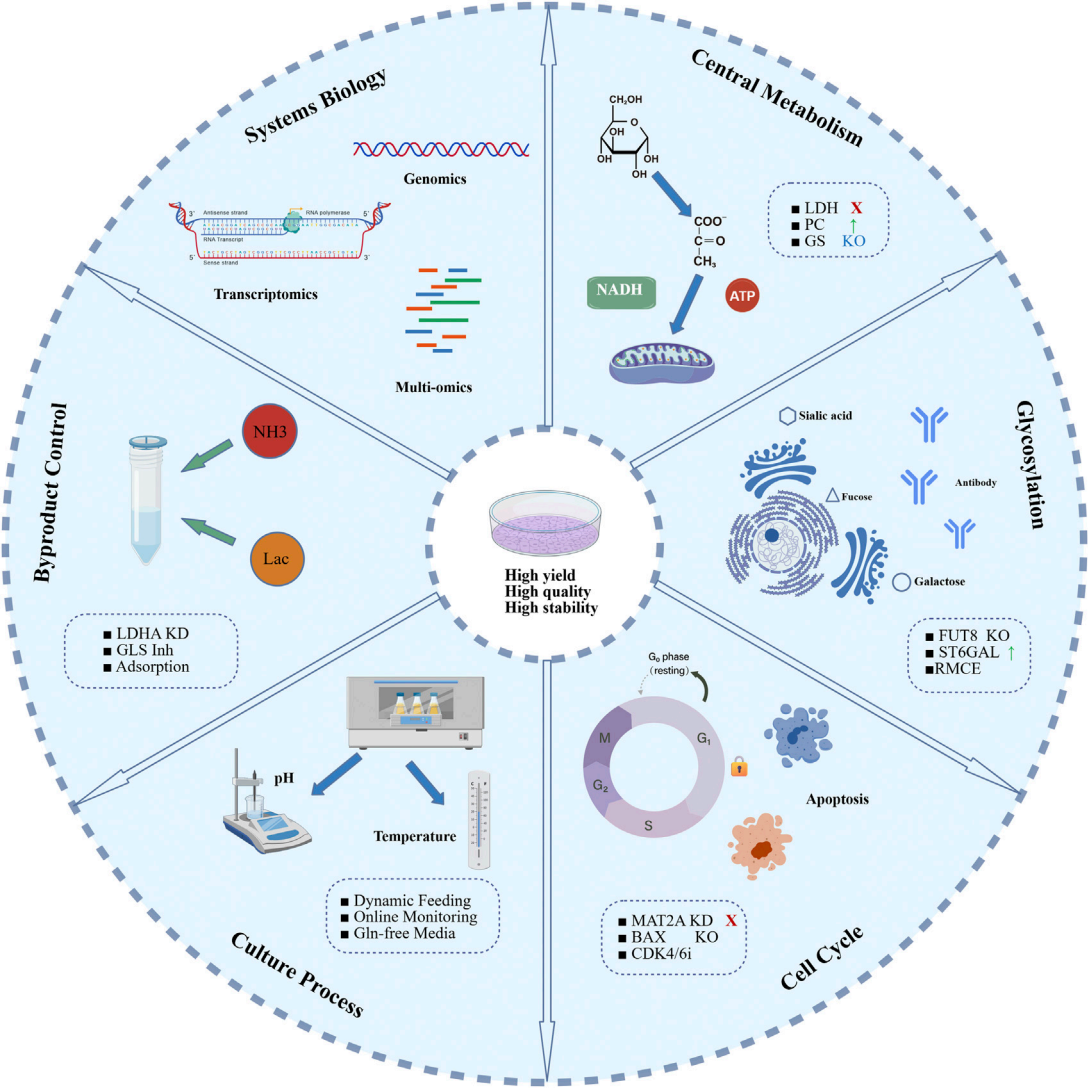
Overview of metabolic engineering strategies for improving recombinant protein production in CHO cells. The schematic diagram displays six core research modules, including central metabolic pathway engineering, glycosylation engineering, cell cycle regulation, culture condition optimization, byproduct accumulation control, and systems biology-assisted rational design. Created with BioGDP.com (Jiang et al., 2025).

This review systematically summarizes recent progress in CHO cell metabolic engineering, with emphasis on global regulation of central metabolism, glycosylation engineering, the interplay between cell cycle control and metabolic reprogramming, culture condition-metabolism interactions, byproduct mitigation strategies, and the application of emerging systems biology technologies (Figure 1). By examining these areas, we not only evaluate current strategies for improving cellular performance, product yield, and quality, but also outline persistent challenges including unpredictable off-target effects of multi-gene editing, long-term cell line instability, and the difficulty of scaling lab-scale optimizations to 10,000 L industrial bioreactors, as well as future trends toward intelligent, integrated development. As conventional cell engineering converges with modern computational biology, metabolic engineering continues to provide essential innovation for efficient and robust biopharmaceutical manufacturing.

## 2 Effect of central metabolic pathways on CHO cell metabolism

Central metabolic pathways—comprising glycolysis, the tricarboxylic acid (TCA) cycle, and the pentose phosphate pathway (PPP)—form the core network governing CHO cell metabolism (Ahn and Antoniewicz, 2011; Kim et al., 2012). Together, they establish the fundamental framework for energy metabolism, redox homeostasis, and biosynthetic precursor supply. Beyond providing essential ATP, NAD(P)H, and carbon skeletons for cell proliferation and protein synthesis, these pathways directly influence the yield, quality, and consistency of recombinant proteins. Glycolysis drives initial glucose breakdown and ATP generation; the TCA cycle further oxidizes pyruvate to produce substantial reducing equivalents; and the PPP supplies ribose-5-phosphate for nucleotide synthesis and NADPH for antioxidant defense and reductive biosynthesis.The coordinated function of these pathways is vital for maintaining metabolic balance under high-density culture conditions. Consequently, a deep understanding and precise regulation of central metabolism have become central to advancing CHO cell metabolic engineering, directly affecting the construction, screening, and performance of engineered cell lines.

### 2.1 Foundational metabolic engineering strategies: gene overexpression, knockdown, and selection systems

Early metabolic engineering efforts concentrated on optimizing core energy metabolism. In batch cultures, CHO-K1 cells typically undergo distinct metabolic phases: high glycolytic flux and lactate accumulation coupled with active TCA cycling during exponential growth, followed by potential lactate re-utilization and overall downregulation of glycolysis and TCA flux in stationary phase (Ahn and Antoniewicz, 2011). This metabolic transition correlates closely with production performance. Cells unable to shift from lactate production to consumption often display weaker TCA activity and lower energy metabolism efficiency, ultimately impairing recombinant protein yield and quality (Gopalakrishnan et al., 2024).

Regulating flux distribution at the pyruvate node is particularly important. Increasing the flux ratio of pyruvate dehydrogenase (PDH) to pyruvate kinase (PK) promotes pyruvate entry into the TCA cycle rather than its reduction to lactate, improving glucose-carbon utilization and decreasing lactate accumulation (Torres et al., 2025). This not only enhances energy efficiency but also reduces growth-inhibiting byproducts. Genetic strategies frequently target key enzymes at this node. For example, overexpressing pyruvate carboxylase (PC), which catalyzes the conversion of pyruvate to oxaloacetate to replenish TCA cycle intermediates, strengthens anaplerosis (the metabolic process that replenishes TCA cycle intermediates consumed for biosynthesis), helping maintain TCA intermediate pools to meet biosynthetic demands. However, such modifications can introduce a substantial metabolic load and may require stage-specific fine-tuning. Stable CHO-K1 cell lines overexpressing PC have demonstrated 20% higher sustained cell density and 15% higher antibody titer in 14-day perfusion cultures, along with 35% reduced lactate accumulation, indicating greater metabolic robustness (Yallop et al., 2003; Gupta et al., 2017). These effects may vary depending on the specific CHO clone and culture conditions.

Imbalances in trace elements, such as copper deficiency, can also impair central glycolytic enzyme activity (e.g., GAPDH), weaken PPP flux, disrupt redox balance, and trigger metabolic reprogramming and stress responses (Nargund et al., 2015). These findings underscore the need to sustain metabolic equilibrium and highlight new potential engineering targets.

A key role of central metabolism is to sustain energy (ATP) and reducing-power (NADH/NAD^+^, NADPH) homeostasis. Under stress conditions like elevated pCO_2_, mitochondrial electron transport chain function becomes constrained, leading to inadequate NAD^+^ regeneration, an increased NADH/NAD^+^ ratio, and subsequent inhibition of TCA flux with concomitant glycolysis upregulation. This ultimately promotes lactate accumulation and disrupts energy metabolism (Brunner et al., 2018). Such metabolic states can also alter the post-translational modification profiles of recombinant proteins.

Engineering solutions often aim to optimize cofactor regeneration—for instance, by enhancing expression of electron transport chain components or introducing exogenous transhydrogenases or dehydrogenases to rebalance NAD(P)H/NAD (P^+^) pools. Notably, copper deficiency not only compromises electron transport, forcing ATP generation via glycolysis, but also increases lactate output to regenerate NAD^+^, thereby mitigating reductive stress (Nargund et al., 2015). Moreover, although upregulating glycolytic enzymes (e.g., LDH, PK) may accelerate growth, it risks perturbing NADPH supply–demand balance, potentially affecting reductive processes such as disulfide bond formation during post-translational modification, as demonstrated in a comparative metabolomic analysis of engineered CHO clones (Wilkens and Gerdtzen, 2015). These observations reinforce the importance of integrated cofactor management in metabolic engineering.

Selection systems rooted in central metabolism remain essential tools for generating recombinant CHO cell lines. The dihydrofolate reductase (DHFR) and glutamine synthetase (GS) systems apply metabolic pressure on nucleotide synthesis and nitrogen metabolism, respectively. This selection pressure enriches cells with stable genomic integration of the transgene and, in many cases, increased transgene copy number; it does not eliminate all low-copy clones, but rather creates a survival advantage for clones with higher and more stable expression. The resulting polyclonal pool still contains copy number heterogeneity, and subsequent single-cell cloning is required to obtain uniformly high-expressing cell lines in industrial CHO host cell platforms (Yang et al., 2022). While enabling efficient transgene amplification and expression, these systems can substantially reshape host-cell metabolism, altering nucleotide pools and amino acid balance.

The DHFR system inhibits folate metabolism to exert selective pressure, whereas the GS system selects for glutamine synthesis capacity. Although both are widely employed across CHO cell lineages, their metabolic consequences require careful evaluation. DHFR-based selection may reprogram nucleotide metabolism, while GS-based selection can influence amino acid homeostasis and energy metabolism. Thus, metabolic side effects must be weighed during cell-line development to identify the most suitable system for a given production need.

While both DHFR and GS selection systems are used in academia, glutamine synthetase knockout (GS-KO) platforms have become the *de facto* industry standard for modern biopharmaceutical manufacturing, and are widely adopted for the production of most newly approved monoclonal antibodies (Barnard et al., 2015).

GS-KO platforms offer several key advantages over traditional DHFR systems. First, they eliminate the need for toxic selection agents such as methotrexate, which can induce genetic mutations and metabolic stress. Second, GS-KO cells cannot survive in glutamine-free media unless they integrate the GS expression cassette along with the transgene, resulting in extremely high selection stringency and low background growth. Third, GS-KO cells exhibit inherently lower ammonia accumulation compared to wild-type CHO cells, as they cannot deaminate glutamine via endogenous GS. This reduces the metabolic burden of ammonia detoxification and improves glycosylation consistency during late-stage culture (Barnard et al., 2015).

Recent advances have further optimized GS-KO platforms through metabolic engineering. For example, overexpression of glutamate dehydrogenase (GDH) in GS-KO cells enhances ammonium assimilation, reducing extracellular ammonia levels by an additional 40% in fed-batch cultures (Srila et al., 2023).

In recent years, novel metabolic selection markers and dual metabolic screening systems based on amino acid and nucleotide biosynthesis pathways have been developed to reduce basal metabolic burden and enhance screening efficiency in CHO cell engineering (Ha et al., 2023). Concurrently, marker-free strategies, such as fluorescence-activated cell sorting based on cell size or intracellular product accumulation, offer alternatives to lower metabolic burden and sustain growth stability. However, these next-generation platforms still face challenges including lower selection stringency compared to traditional GS-KO systems, higher development costs, and limited industrial validation at large scale (Webster et al., 2021).

### 2.2 Advanced metabolic engineering: dynamic regulation, transcription factor engineering, and systems metabolic reprogramming

Advances in synthetic and systems biology have shifted metabolic engineering from single-enzyme manipulation toward network-level, dynamic, and precise interventions.

Metabolite-sensor-based dynamic control, as a representative class of synthetic gene regulatory systems, represents an emerging approach for optimizing energy metabolism and recombinant protein production performance in CHO cells. The development of rationally designed synthetic gene circuits has been widely recognized as a transformative direction for mammalian cell metabolic engineering, enabling sophisticated, autonomous regulation of cellular behaviors beyond the capabilities of conventional static genetic modification strategies (Xie and Fussenegger, 2018). Conventional static metabolic engineering strategies relying on constitutive overexpression or knockdown often suffer from unpredictable metabolic burden and inflexible flux allocation across different culture phases. Rationally designed synthetic gene control circuits can effectively mitigate these limitations by enabling real-time, autonomous regulation of target gene expression. A variety of engineered circuit architectures, including incoherent feedforward circuits, negative feedback circuits and hybrid integrated designs, have been developed to improve expression robustness against gene dosage variation and reduce excessive consumption of endogenous cellular resources. In CHO cell-based protein manufacturing systems, these optimized synthetic control circuits have been demonstrated to achieve up to a 2.6-fold improvement in recombinant protein yield compared with standard expression systems (Lillacci et al., 2018).

The core design logic of such dynamic regulation strategies can be extended to central carbon metabolism engineering: by constructing metabolite-responsive genetic circuits that sense key intracellular metabolites such as lactate or the NADH/NAD^+^ ratio, the expression of key rate-limiting enzymes in glycolysis and the TCA cycle can be dynamically adjusted in real time. This enables cells to autonomously reshape carbon metabolic flux distribution according to dynamically changing culture environments, reduce the accumulation of growth-inhibitory by-products, and maintain metabolic stability during long-term high-density fed-batch culture.

A key application value of this dynamic metabolic regulation paradigm is to decouple cell growth from recombinant protein production, a strategy that has attracted increasing attention in industrial biomanufacturing. By separating the culture process into a dedicated growth phase (for accumulating high viable cell density) and a production phase (for redirecting cellular resources toward target protein synthesis), this strategy is particularly effective for improving the expression of difficult-to-express next-generation biotherapeutics such as bispecific antibodies and fusion proteins. Compared with traditional proliferation control methods including temperature shifts and chemical induction agents—which often act on multiple non-specific pathways and lead to variable impacts on product quality and long-term culture viability—synthetic biology-based targeted dynamic regulation offers a more precise and controllable technical alternative (Donaldson et al., 2021).

This strategy marks an important paradigm shift from static, constitutive single-gene modification to dynamic, environment-adaptive feedback regulation of metabolic networks, and provides a feasible technical blueprint for the construction of next-generation intelligent, high-efficiency CHO cell factories.

Transcription factor (TF) engineering has emerged as a powerful means of orchestrating cellular metabolism systemically. By modulating the expression or activity of key TFs, multiple pathways can be coordinated to simultaneously enhance target-product synthesis and cellular stress tolerance. ChREBP and HIF-1α target glycolytic pathway genes including hexokinase, phosphofructokinase and pyruvate kinase to globally regulate glucose metabolic flux; XBP1s and ATF6 regulate endoplasmic reticulum (ER) chaperone expression and secretory pathway capacity to improve protein folding efficiency; Activating transcription factor 4 (ATF4) coordinates amino acid metabolism and redox homeostasis to enhance cellular stress resistance under nutrient limitation. Co-overexpression of BLIMP1 and XBP1s, for example, synergistically elevates secretory pathway flux and increases recombinant protein yield (Torres and Dickson, 2022). Engineering eukaryotic translation initiation factor 3 (eIF3) subunits can indirectly influence metabolic state and growth through translational reprogramming (global regulation of protein synthesis rates and specificity) (Roobol et al., 2020). A major advantage of TF engineering is its ability to regulate suites of functionally related genes concurrently, thereby achieving synergistic network optimization. Overexpression of a glycolysis-related transcription factor such as carbohydrate response element binding protein (ChREBP) or hypoxia-inducible factor 1-alpha (HIF-1α) can simultaneously upregulate multiple rate-limiting glycolytic enzymes (e.g., hexokinase, phosphofructokinase, pyruvate kinase), in CHO-K1 and CHO-S host cells, avoiding the metabolic imbalances that may arise from modifying single enzymes (Gutierrez-Gonzalez et al., 2019). Similarly, ATF4 overexpression helps CHO-S cells maintain redox and energy homeostasis under nutrient limitation, facilitating the selection of perfusion-compatible lines with improved viability and stable production (Chang et al., 2022). Such approaches illustrate a paradigm shift from single-enzyme to network-level metabolic programming. This enables more robust and predictable control over cellular metabolism and improves resource allocation for recombinant protein synthesis.

The endoplasmic reticulum (ER) unfolded protein response (UPR) consists of three parallel signaling arms—inositol-requiring enzyme 1 (IRE1)/X-box binding protein 1 spliced (XBP1s), activating transcription factor 6 (ATF6), and protein kinase RNA-like ER kinase (PERK)—that collectively maintain secretory pathway homeostasis. While XBP1s overexpression is the most widely used strategy to enhance antibody secretion, the ATF6 and PERK pathways play equally critical but underappreciated roles in CHO cell engineering.

ATF6, a membrane-bound transcription factor, is activated by ER stress and translocates to the nucleus to upregulate chaperone genes, including BiP (binding immunoglobulin protein, also known as 78 kDa glucose-regulated protein, GRP78) and GRP94 (94 kDa glucose-regulated protein). Dickson and colleagues demonstrated that overexpression of the active form of ATF6α in CHO-K1 cells led to a 1.5-fold increase in recombinant antibody secretion during fed-batch cultures, without significantly impacting cell growth (Torres and Dickson, 2022). This effect was attributed to enhanced ER folding capacity, rather than increased transgene transcription.

The PERK pathway exerts dual effects on recombinant protein production: moderate PERK activation transiently attenuates global protein synthesis to reduce ER load and upregulates antioxidant genes, which can help maintain cell viability in late-stage culture and reduce misfolded protein aggregates (Ron and Walter, 2007). However, sustained PERK activation also decreases specific productivity via global translation suppression (Castellano et al., 2023). Therefore, multi-arm UPR engineering (e.g., combined XBP1s overexpression and ATF6 activation) can achieve synergistic enhancement of secretory capacity by balancing protein folding efficiency and synthesis rate (Bommiasamy et al., 2009).

Beyond direct TF manipulation, engineering upstream signaling pathways offers additional promise. Modulating energy- and nutrient-sensing cascades such as AMPK or mTORC1 can globally reset cellular metabolic patterns, improving resource allocation toward product synthesis under limiting conditions. For example, mTORC1 activation enhances global translation rates and redirects carbon flux from glycolysis to oxidative phosphorylation, increasing ATP availability for protein synthesis; AMPK activation promotes autophagy to recycle cellular components and maintain energy homeostasis during nutrient limitation. Engineering mTORC1 signaling, in particular, has been shown to enhance both cell growth and recombinant protein production in CHO cells by coordinating translation and metabolism (Chang et al., 2022). Engineering key nodes in stress-response pathways—including the unfolded protein response and oxidative stress defense—further enhances metabolic adaptability and survival under industrial culture stresses (Ron and Walter, 2007).

### 2.3 Artificial intelligence and multi-omics integration: data-driven metabolic phenotyping and rational design

Metabolic Flux Analysis (MFA) is essential for quantitatively mapping intracellular metabolic states. Conventional mass-balance methods estimate fluxes from substrate uptake and product secretion rates but struggle to resolve parallel or cyclic pathways, such as the pentose phosphate pathway. ^13^C-labeling techniques, particularly ^13^C-MFA, overcome this by tracking labeled carbon atoms through the metabolic network, providing additional constraints that greatly improve the spatiotemporal resolution and accuracy of flux estimates (Reddy et al., 2023). This approach helps pinpoint metabolic bottlenecks under different culture regimes, guiding targeted engineering interventions.

Beyond flux analysis, absolute metabolite quantification aids in evaluating reaction thermodynamics, while integration of transcriptomic and proteomic data unveils the molecular underpinnings of metabolic regulation, offering multi-layered evidence for target selection (Matsuda et al., 2017). Progress in analytical tools—including fluorescent protein sensors for real-time metabolite tracking—now supports the dynamic control of metabolic pathways. Together, these techniques provide a system-level perspective on metabolic phenotypes across culture stages and after genetic modifications.

Recently, coupling untargeted metabolomics with machine learning has opened new routes for high-throughput screening of engineered cell lines with desired metabolic traits. Untargeted metabolomics combined with machine learning enables high-throughput mining of metabolic biomarkers closely associated with high-productivity CHO phenotypes, providing guidance for rational cell engineering design (Kavoni et al., 2025). AI models, including deep learning and random forests, can integrate multi-omics datasets (metabolomics, transcriptomics, fluxomics) to predict metabolic engineering targets and simulate phenotype outcomes under various modification strategies, substantially accelerating design cycles and success rates. For example, machine learning applied to dynamic metabolite profiles from CHO cultures has successfully predicted enzyme combinations critical for lactate metabolic switching, enabling the construction of stable, high-yield cell lines with efficient lactate clearance in high-density culture (Kavoni et al., 2025).

### 2.4 Metabolic engineering for high-yield and high-quality recombinant protein production: from pathway optimization to organelle engineering

The glycosylation of recombinant proteins critically depends on nucleotide sugars and energy supplied by central metabolism, whose availability directly impacts glycosylation efficiency and homogeneity (Wong et al., 2010).

In summary, from foundational energy-metabolism tuning and selection systems to advanced dynamic control and transcriptional reprogramming, and onward to data-driven rational design, a deep understanding and precise manipulation of central metabolic pathways underpin the entire CHO cell metabolic engineering workflow. This integrated approach remains central to elevating cellular performance and enabling the high-quality, high-yield production of recombinant proteins.

## 3 Glycosylation metabolic engineering

Glycosylation constitutes one of the most critical quality attributes of recombinant therapeutic proteins, with direct impacts on clinical efficacy, safety, immunogenicity, *in vivo* half-life, and effector functions. Core fucosylation level of antibody Fc region is the key regulator of antibody-dependent cellular cytotoxicity (ADCC), and afucosylated antibodies exhibit 10–100 fold enhanced ADCC activity. In terms of pharmacokinetic properties, terminal sialylation extends serum half-life by reducing clearance through the asialoglycoprotein receptor. From a safety perspective, non-human glycan epitopes such as Neu5Gc can trigger immune responses in patients, leading to adverse reactions and reduced drug efficacy. Additionally, glycan structures protect proteins from proteolytic degradation and maintain correct spatial conformation, which is essential for protein stability. Although CHO cells inherently perform human-like glycosylation, the resulting glycan profiles are modulated by a multitude of factors, including host genetic background, protein sequence, culture environment, and medium composition (Kalkan et al., 2023; Li et al., 2024). Glycosylation engineering seeks to precisely control glycan structures commonly found in CHO-derived therapeutic proteins, including high-mannose glycans, core-fucosylated complex glycans (G0F, G1F, and G2F), and sialylated complex glycans, through integrated genetic and bioprocess interventions, thereby optimizing the functional characteristics of therapeutic proteins (Kotidis et al., 2023). With growing mechanistic insight and technological advancement, the field has progressed from traditional process optimization to a stage of precision regulation, driven by targeted gene editing of glycosylation-related genes (e.g., *FUT8*, *CMAH*, *B4GALT1*, *ST6GAL1*) and systems biology (Amann et al., 2019). The goal is not only to achieve target-designed ideal glycan profiles tailored to therapeutic mechanisms, such as afucosylated glycoforms for enhanced ADCC of antitumor antibodies, highly sialylated glycoforms for prolonged serum half-life of fusion proteins, and fully humanized glycoforms to minimize immunogenicity (Li et al., 2022) but also to engineer stable cell lines capable of consistently producing proteins with superior quality attributes.

### 3.1 Genetic strategies for glycosylation regulation

Glycosylation is an energy- and precursor-intensive process that draws both ATP and nucleotide sugar donors directly from central metabolic pathways. Each glycosylation event consumes ATP and sugar donors whose supply is tightly regulated by glycolysis, the PPP, and the TCA cycle. Rational redesign of central metabolism to optimize precursor and energy provision is therefore fundamental to achieving consistent, high-quality glycan structures in biopharmaceuticals. Engineering the flux through these pathways can significantly improve glycosylation profiles, thereby enhancing biologic efficacy and safety.

Protein secretion efficiency is likewise governed by metabolic status. Folding and trafficking in the ER and Golgi apparatus are energy-demanding processes. Boosting the ATP-supplying capacity of central metabolism for these organelles, or engineering chaperone expression (e.g., BiP) to relieve ER load, can effectively increase secretory flux (Preissler et al., 2015).Concurrently, modulating autophagy flux—a conserved degradation process that recycles damaged organelles and misfolded proteins—helps clear ER-associated antibody aggregates, maintaining an unobstructed secretory pathway.

Mitochondrial function engineering is gaining attention as a means to support production. Overexpression of mitochondrial fusion proteins (e.g., MFN1/MFN2) or optimized mitophagy promotes a healthy, functional mitochondrial network, sustaining efficient oxidative phosphorylation and ATP supply (Galluzzi et al., 2018).Engineering mitochondrial membrane transporters to improve metabolite shuttling—such as mitochondrial pyruvate carriers (MPC1/2) for pyruvate and the malate-aspartate shuttle components for malate—further enhances central metabolic efficiency (Son et al., 2015; Junghans et al., 2019; Bulte et al., 2020).Additionally, cytoskeleton dynamics—closely tied to vesicular transport—rely on continuous ATP provision. Thus, supporting cytoskeletal function indirectly through energy-metabolism optimization also represents a viable secretion-promoting strategy.

Systematic redesign of glycosylation pathways in CHO cells is routinely achieved via gene editing and overexpression. For instance, CRISPR/Cas9-mediated knockout of fucosyltransferase 8 (FUT8) markedly reduces antibody fucosylation, leading to enhanced antibody-dependent cellular cytotoxicity (ADCC) (Wang and Betenbaugh, 2018; Lancho et al., 2026). Similarly, multiplex gene-editing strategies—such as simultaneously disrupting ten N-glycosylation-related genes while introducing human beta-galactoside alpha-2,6-sialyltransferase 1 (ST6GAL1)—can substantially increase the proportion of desired glycoforms (e.g., A2G2S2, a fully processed bi-antennary glycan with two galactose and two sialic acid residues), yielding glycosylation profiles that more closely resemble those of human proteins (Amann et al., 2019). Engineered lines developed in this manner have demonstrated stable glycosylation and high product titers over extended passages, underscoring the potential of coordinated multi-gene editing for constructing high-yield, glycoform-consistent cell lines.

Targeted knockout or downregulation of *FUT8* is the most classic strategy to completely eliminate core fucosylation, and is widely used for development of anti-tumor antibodies requiring maximum ADCC enhancement. In contrast, overexpressing the GDP-fucose transporter solute carrier family 35 member C1 (SLC35C1) and related biosynthetic enzymes is a strategy to increase core fucosylation levels, applied when target therapeutic proteins require high and stable fucosylation to avoid excessive immune activation. These two approaches represent opposite directions of glycan fine-tuning for different clinical development goals, providing flexible regulatory tools for glycoengineering (Yamane-Ohnuki et al., 2004; Malphettes et al., 2010). Site-specific integration (SSI) strategies, particularly recombinase-mediated cassette exchange (RMCE) and landing pad systems, have become the industrial gold standard (Carver et al., 2020). The Genentech targeted integration (TI) platform uses a single pre-validated genomic landing pad to achieve highly consistent transgene expression across independent clones. Compared with random integration-based cell line development, this platform reduces the coefficient of variation (CV) of batch-to-batch antibody titer and core fucosylation level by more than 70%. The measurement is performed by quantifying end-of-fed-batch culture titer and glycan composition from dozens of parallel monoclonal cell lines, then calculating and comparing CV values between the two development methods (Carver et al., 2020). Targeted disruption of functional genes such as *CMAH* (cytidine monophosphate-N-acetylneuraminic acid hydroxylase) is a dedicated glycoengineering strategy to eliminate production of the immunogenic non-human glycan N-glycolylneuraminic acid (Neu5Gc) in CHO cells. Separately, industrial cell line development platforms typically integrate the therapeutic transgene into well-characterized transcriptionally active genomic loci (e.g., safe harbor sites) to maximize and stabilize recombinant protein productivity. These two approaches are complementary genomic engineering strategies: one optimizes product quality attributes, the other enhances production performance. Modern industrial chassis cells often combine both modifications: transgene integration at transcriptionally active sites, plus *CMAH* and other glyco-gene knockouts, to achieve both high yield and controlled, high-quality glycoforms (Zhang et al., 2023; Cao et al., 2025). Epigenetic modulation, including the use of histone deacetylase inhibitors (iHDACs), can alter glycosylation profiles through changed expression of glycosylation-related genes (Nmagu et al., 2021; Chang et al., 2022). Meanwhile, knockout of epigenetic regulators such as Bcor (BCL6 corepressor) can mitigate the growth inhibition caused by iHDACs, allowing higher cell densities without compromising specific productivity (Kim et al., 2024). Together, these genetic tools provide a robust foundation for precise control over therapeutic protein glycosylation.

Recent advances have particularly focused on the core fucosylation pathway. *FUT8* knockout is the classic strategy to completely eliminate core fucosylation, as first demonstrated in CHO-DG44 cells (Yamane-Ohnuki et al., 2004); in addition, knocking down or knocking out the GDP-fucose transporter *SLC35C1*, or depleting the intracellular GDP-fucose pool by targeting its biosynthetic enzymes (e.g., FX encoded by GDP-mannose 4,6-dehydratase *(TSTA3)*), can also reduce core fucosylation and increase the proportion of afucosylated antibodies in CHO-K1-derived host cells (Malphettes et al., 2010; Tejwani et al., 2018).

FUT8 knockout completely eliminates the core fucosylation reaction, achieving >99% afucosylated antibodies with 10–100 fold enhanced ADCC activity; this strategy remains the gold standard for industrial production of high-ADCC therapeutic antibodies, and the engineered cell lines can maintain robust growth and titers up to 5.2 g/L in perfusion mode (Yamane-Ohnuki et al., 2004). In contrast, *SLC35C1* knockdown only partially reduces GDP-fucose transport into the Golgi, limiting substrate availability for FUT8 and typically achieving 60%–80% fucosylation reduction with minimal impact on cellular growth and fitness, making it suitable for applications requiring moderate ADCC enhancement (Malphettes et al., 2010; Tejwani et al., 2018).

Combined multi-gene editing strategies, such as simultaneous *FUT8* knockout and *CMAH* disruption, can eliminate both core fucosylation and the immunogenic non-human Neu5Gc glycan in a single engineering step, further improving antibody safety and effector function in CHO-S and CHO-K1-based glycoengineered chassis cells (Amann et al., 2019; Zhang et al., 2023).

Although many modern industrial CHO host strains exhibit relatively low endogenous CMAH activity and produce only trace levels of Neu5Gc under standard culture conditions, targeted *CMAH* disruption remains a highly relevant engineering strategy for multiple key reasons. First, Neu5Gc incorporation can be elevated under specific culture conditions such as when using animal-derived media components or under metabolic stress, and complete *CMAH* knockout eliminates this variability to ensure batch-to-batch quality consistency. Second, for therapeutic proteins intended for long-term or repeated *in vivo* administration, even trace levels of non-human glycan epitopes carry potential immunogenic risks, and *CMAH* ablation provides an additional safety margin to meet stringent regulatory requirements. Third, *CMAH* knockout serves as a foundational modification for building fully humanized glycoengineered chassis cells, which can be further combined with other glycoengineering modifications to produce custom-defined glycoforms (Cao et al., 2025).

### 3.2 Effect of host cell line and clonal variation on glycosylation

Different CHO host lineages (e.g., CHO-K1, CHO-DG44) exhibit distinct glycosylation capacities and gene expression profiles owing to divergent genetic backgrounds (Li et al., 2024). These differences manifest in the expression levels and substrate specificities of glycosyltransferases and glycosidases, as well as in the availability of intracellular nucleotide-sugar donors. Significant glycoform heterogeneity also exists among individual clones, necessitating combined transcriptomic and glycan screening during cell-line development to isolate clones with desired glycosylation traits (Konitzer et al., 2015). Notably, the glycosylation state of the cell surface can serve as an early predictor of the glycan profile on secreted proteins; techniques such as lectin staining therefore enable preliminary glycan profiling and clone screening (Kotidis et al., 2023). Integrating lectin staining with high-throughput productivity screening allows rapid identification of “high-yield, high-quality” clones from large candidate pools, greatly improving cell-line construction success.

Coupling single-cell cloning with microfluidic sorting further streamlines the isolation of clones with target glycosylation features, accelerating cell-line development. A thorough understanding of glycosylation variation across host lines and clones is essential for rational selection of production cell lines and for defining appropriate glycan control strategies.

Single-cell analysis technologies are now revealing the molecular foundations of glycosylation heterogeneity within and between clones. Single-cell RNA sequencing (scRNA-seq) resolves discrete expression patterns of glycosylation-related genes across a population, linking transcriptional signatures to final product glycoforms. Mass spectrometry-based single-cell secreted protein analysis directly measures antibody glycosylation from individual cells, offering unprecedented resolution for early identification of clones with optimal glycoform homogeneity. Applying these tools, researchers have successfully isolated and expanded elite single clones from mixed populations. Such clones maintain high growth rates and product titers in both shake-flask and bioreactor cultures while exhibiting less than 5% batch-to-batch variation in critical glycan attributes (e.g., fucosylation level) (Kotidis et al., 2023), achieving concurrent gains in yield and quality consistency. High-throughput single-cell methodologies thus enable precise selection of “premium” clones that meet stringent quality specifications from complex cellular pools. The emergence of automated microscale bioreactor and optofluidic platforms has further revolutionized industrial cell line development by integrating high-throughput cultivation, functional screening, and single-cell isolation into a single workflow. The ambr15/250 system (Sartorius) enables parallel cultivation of up to 250 independent clones in 15 mL bioreactors with fully automated feeding and process control (Wiegmann et al., 2021), reproducing fed-batch performance at microscale with <13% titer variation compared to 5 L bioreactors and reducing clone screening timelines by 50% (Amanullah et al., 2010; Wiegmann et al., 2021). The Berkeley Lights Beacon platform, based on optofluidic single-cell manipulation, allows real-time monitoring of antibody secretion from thousands of individual cells in parallel, enabling direct selection of high-productivity clones with desired glycosylation profiles within 3 days and providing >99% clonality assurance (Le et al., 2020). These technologies have become the *de facto* standard for modern biopharmaceutical cell line development, enabling the screening of tens of thousands of clones per campaign while maintaining strict quality control.

### 3.3 Effect of culture environment and metabolites on glycosylation

Culture conditions, such as elevated ammonia concentration, can profoundly impact glycosylation. High ammonia levels not only inhibit cell growth but also increase glycoprotein microheterogeneity, with the most pronounced effects being reduced sialylation and galactosylation (Yang and Butler, 2000). This phenomenon is attributed to ammonia-induced alkalinization of the Golgi lumen and subsequent suppression of glycosyltransferase activity. Supplementation of exogenous components, such as CHO-derived microsomes, has been shown to partially restore glycosylation efficiency and product yield (Gurramkonda et al., 2018). Additionally, careful control of metabolic byproducts (e.g., lactate, ammonia) and key culture parameters—including pH, dissolved oxygen, and pCO_2_—is essential to sustain glycosyltransferase activity and maintain a balanced nucleotide sugar donor pool. Dissolved CO_2_ influences intracellular pH, which in turn affects Golgi-resident enzyme activities. While temperature shift strategies can boost protein titer, they may compromise glycosylation efficiency and thus require careful optimization to balance yield and quality. Similarly, manganese ions, essential cofactors for many glycosyltransferases, must be precisely regulated to avoid adverse effects on glycan quality.

The mechanistic links between metabolic byproducts and glycosylation are increasingly understood. Excessive glycolysis and lactate accumulation disrupt intracellular nucleotide sugar homeostasis, limiting the supply of UDP-GlcNAc and other key glycosylation precursors in fed-batch CHO cultures (Fan et al., 2015). Elevated ammonia alters Golgi luminal pH and suppresses the activity and transport function of CMP-sialic acid transporter, markedly reducing terminal sialylation of recombinant proteins in CHO cells (Son et al., 2011; Lin et al., 2015). To address these bottlenecks, process-engineering strategies—including dynamic nutrient feeding (e.g., controlled glucose and glutamine supplementation) and *in situ* metabolite removal (e.g., adsorbents, perfusion culture)—have proven effective in minimizing lactate and ammonia accumulation, thereby stabilizing glycan profiles. For example, an optimized perfusion process using a clonal CHO-K1 cell line with online metabolite monitoring enabled stable viable cell density and product titer for over 60 days while keeping lactate and ammonia below critical thresholds. The resulting product exhibited a 30% increase in sialylation compared with batch culture, along with markedly improved batch-to-batch consistency (Karengera et al., 2018), demonstrating a robust process-cell line pairing suitable for long-term continuous manufacturing.

### 3.4 Model-guided glycosylation engineering

To improve the rationality and predictive power of glycosylation control, computational tools such as CHOGlycoNET have been developed. This model, constructed by Kotidis et al. (2023), integrates 200+ experimental glycan datasets and simulates the complete CHO glycosylation reaction network, identifying critical regulatory nodes such as UDP-GlcNAc availability and Golgi pH (Kotidis et al., 2023). Such systems-biology approaches facilitate a holistic understanding of glycosylation regulation and propel the field toward precision glycoengineering. Kinetic models can forecast shifts in glycan distribution under varying culture conditions, while constraint-based models pinpoint key factors limiting glycosylation efficiency. Machine-learning methods are increasingly applied to construct predictive models of glycoforms by leveraging historical culture data, enabling accurate forecasts of final-product glycosylation traits. Combining *in silico* predictions with experimental validation creates a design-build-test-learn cycle that substantially accelerates glycosylation engineering. Multi-scale integration further allows accurate prediction and optimization of glycosylation outcomes across scales—from metabolic reactions to population-level behavior.

With growing volumes of multi-omics data, machine learning and artificial intelligence offer powerful avenues for glycoform prediction. Regression models and neural networks trained on process parameters (e.g., nutrient and metabolite concentrations), cell physiological states (e.g., viability, size), and transcriptomic features can deliver high-accuracy predictions of critical glycan attributes such as fucosylation and sialylation levels (Li et al., 2022). Furthermore, coupling kinetic models of glycosylation pathways with steady-state models of central carbon metabolism and nucleotide sugar synthesis yields integrated “metabolism-glycosylation” frameworks. Such combined models simulate how genetic modifications (e.g., gene overexpression) or environmental shifts alter precursor availability and energy status to influence glycan assembly, thereby guiding synergistic metabolic and glycosylation engineering strategies. In one study guided by the CHOGlycoNET network model, model-informed multi-target metabolic engineering was applied. This strategy included optimization of glycolytic flux distribution and overexpression of two key rate-limiting nucleotide sugar synthases: UDP-N-acetylglucosamine pyrophosphorylase 1 (UAP1) and GDP-mannose 4,6-dehydratase (GMDS). It not only doubled the proportion of the target G0F glycoform but also unexpectedly enhanced cellular energy metabolism in industrial CHO host cell lines. The engineered lines achieved a 25% higher peak viable cell density and a 40% increase in final antibody titer in fed-batch culture (Kotidis et al., 2023), accompanied by improved metabolic robustness. This example underscores the significant value of model-guided rational design in developing high-performance cell lines with superior yield, stability, and controllable glycoforms.

Glycosylation engineering in CHO cells encompasses a multifaceted approach integrating genetic, cellular, process, and computational strategies. Genetic modifications, such as targeted gene editing and overexpression, enable precise control over glycan structures to enhance protein functionality. The inherent variability among host cell lines and clones necessitates careful screening and selection to ensure consistent glycan profiles. Furthermore, culture conditions and metabolite levels significantly impact glycosylation efficiency, requiring optimized process control to maintain product quality. Advancing these efforts, model-guided and AI-driven approaches now support the rational design of glycosylation pathways, linking metabolic states to glycan outcomes. Together, these integrated strategies facilitate the development of robust CHO cell platforms capable of producing recombinant proteins with desired glycosylation attributes (Table 2).

**TABLE 2:** Summary of glycosylation engineering strategies.

Strategy	Specific methods	Regulation target	Effects
Genetic modification	FUT8 knockout, SLC35C1 Knockdown/Knockout, ST6GAL1 introduction	Reduce fucosylation, increase sialylation	Enhance ADCC effect; Complete afucosylation with mild growth penalty; Moderate fucosylation reduction with minimal fitness impact
Multi-gene editing	Multi-gene knockout Human gene introduction	Humanized glycoforms, increase A2G2S2 proportion	Glycoforms closer to human, high editing complexity, stability needs verification
Epigenetic regulation	HDAC inhibitors (iHDACs), Bcor knockout	Maintain glycosylation quality, increasing cell density	Accompany growth inhibition, need to balance yield and quality
Culture environment regulation	Control ammonia concentration, add Mn^2+^, Dynamic feeding, temperature shift optimization	Maintain glycosyltransferase activity, precursor supply balance	Strong process controllability, requires real-time monitoring and fine-tuning
Model and AI prediction	CHOGlycoNET model, ML for glycoform prediction, Metabolism-glycosylation coupling models	Rational glycoform design, process parameter optimization	Improve design efficiency, requires substantial data and model validation

### 3.5 Metabolic engineering challenges for bispecific and complex molecules

The growing demand for bispecific antibodies (bsAbs), antibody-drug conjugates (ADCs), and fusion proteins has introduced new metabolic challenges for CHO cell engineering. Unlike conventional monoclonal antibodies, which consist of two identical heavy chains and two identical light chains, complex molecules often require the co-expression of multiple distinct polypeptide chains in precise stoichiometric ratios (Spiess et al., 2015).

Chain imbalance is the most common bottleneck in bsAb production. Excess expression of one chain can lead to the formation of misfolded aggregates, incorrect disulfide bonds, and non-functional byproducts. This imposes a significant burden on the ER and UPR pathways, reducing cell viability and overall productivity (Gutierrez-Gonzalez et al., 2019). Metabolic engineering strategies to address this issue cover multiple dimensions. Signal peptide optimization uses different signal peptides with varying secretion efficiencies for each chain to balance their expression levels. Molecular chaperone engineering achieves folding enhancement through overexpression of ER chaperones such as BiP and PDI (protein disulfide isomerase), which assists the correct assembly of complex polypeptide chains. Amino acid supply optimization focuses on targeted supplementation of amino acids that are disproportionately consumed in complex molecules, such as cysteine and tryptophan, to relieve metabolic bottlenecks caused by unbalanced amino acid consumption in CHO-K1 fed-batch cultures (Fan et al., 2015).

Additionally, complex molecules often require more extensive post-translational modifications, including multiple glycosylation sites and disulfide bonds. This increases the demand for nucleotide sugar precursors and ATP, requiring coordinated engineering of central metabolism and glycosylation pathways in industrial CHO host cell lines (Wong et al., 2010).

## 4 Cell cycle regulation and metabolic reprogramming

In CHO cell cultures, coordinated regulation of the cell cycle and programmed cell death pathways is essential for extending culture longevity, maintaining high viable cell density, and increasing recombinant protein yield. The orderly progression of the cell cycle is governed by a network of key regulators, including cyclins (e.g., Cyclin D1), cyclin-dependent kinases (e.g., CDK4/6), and their inhibitors (e.g., p21, p27). This regulatory framework not only controls entry into the proliferative cycle but also profoundly influences the cell’s overall metabolic state and resource allocation. As cells shift between proliferation and production phases, their metabolic networks undergo extensive adaptive reprogramming. Consequently, targeting cell-cycle checkpoints to modulate metabolism has become a central engineering strategy for improving culture performance and developing high-yield, stable CHO cell lines.

### 4.1 G1 arrest triggers a global metabolic switch from proliferation to production

Inducing G1 phase arrest is a core approach for driving metabolic reprogramming in CHO cells. Studies consistently demonstrate that precise G1 arrest—achieved through physical, chemical, or genetic means—markedly elevates the specific productivity (qP) of recombinant proteins by systematically redirecting cellular resources from biomass synthesis toward recombinant protein production (Tihanyi and Nyitray, 2020). This fate transition entails a global reshaping of the metabolic network, rooted in molecular crosstalk between cell-cycle controllers and metabolic signaling pathways (Kim et al., 2012), with the ultimate aim of engineering cell lines that efficiently convert growth potential into production capacity.

From a metabolic perspective, rapidly proliferating CHO cells display pronounced anabolic traits: high glycolytic flux supports ATP generation and supplies carbon skeletons for biosynthesis; an active pentose phosphate pathway provides ribose-5-phosphate for nucleotides and NADPH for lipid synthesis and redox balance; and TCA cycle intermediates are diverted toward anabolic reactions. Upon G1 arrest, however, the metabolic phenotype shifts substantially. G1-arrested cells typically exhibit downregulated glycolysis and enhanced mitochondrial oxidative phosphorylation (OXPHOS) (Tang et al., 2020), a transition modulated by transcription factors such as HIF-1α (Goda et al., 2003). Mechanistically, reduced lactate accumulation during G1 arrest arises from three coordinated regulatory events that work in concert to reshape cellular metabolic state. Downregulation of G1 cyclins such as Cyclin D1 suppresses the phosphatidylinositol 3-kinase/protein kinase B/mammalian target of rapamycin (PI3K/Akt/mTOR) signaling axis, transcriptionally reducing expression of key glycolytic enzymes including HK2 and LDHA, thus decreasing overall glycolytic flux. Meanwhile, G1 arrest promotes mitochondrial biogenesis and enhances pyruvate dehydrogenase (PDH) complex activity, shunting a larger proportion of pyruvate into the TCA cycle for complete oxidation rather than conversion to lactate. Furthermore, reduced HIF-1α transcriptional activity in G1-arrested cells further downregulates glycolytic gene expression, reinforcing the metabolic shift from glycolysis to oxidative metabolism in industrial fed-batch CHO cell cultures (Gopalakrishnan et al., 2024).

This metabolic rewiring offers dual benefits: oxidative metabolism delivers a more sustained ATP supply for energy-intensive processes like protein synthesis, while reduced lactate accumulation alleviates culture acidification and growth inhibition, facilitating long-term high-density cultivation. The stable establishment of this phenotype often involves homeostatic processes such as autophagy to preserve organelle function.

Genetic evidence strongly supports G1 arrest as a route to high-yield cell lines. RNA interference (RNAi)-mediated silencing of methionine adenosyltransferase 2A (MAT2A), for example, induces G1 arrest by suppressing Cyclin D1 and activating p21, while increasing monoclonal antibody yield ∼1.7-fold compared to the parental cell line in fed-batch culture (Gao et al., 2025), enlarging cell volume, and elevating overall metabolic activity. The resulting engineered line effectively transitions from a proliferative to a productive state, functioning as a metabolically enhanced protein-production platform. Similarly, modulating proto-oncogenes like Myc can concurrently influence cell-cycle progression and glucose metabolism, offering additional targets for designing production-oriented metabolic phenotypes.

Reversible chemical interventions provide equally powerful tools for dynamic bioprocess control. Small-molecule CDK4/6 inhibitors (e.g., Palbociclib) enable precise, reversible G1 arrest and can indirectly modulate mTORC1 signaling to affect protein synthesis rates. Such agents support “grow-then-produce” strategies by allowing rapid biomass accumulation followed by synchronized conversion to a high-production state. Physical methods such as hypothermia are also widely used to induce G1 arrest, acting through altered cyclin expression (Avello et al., 2022). Together, these diverse interventions form a versatile toolkit for systematically boosting CHO cell production through cell-cycle engineering.

Recent advances in genome-wide CRISPR knockout screens have enabled unbiased identification of novel engineering targets. Marzluf et al. performed a genome-wide screen of 17,761 expressed genes in both shake-flask and 3 L perfusion cultures, identifying 235 growth-enhancing knockouts and defining the CHO_EG2025 benchmark set of 3,110 essential fitness genes. This study revealed that knockout of amino acid catabolism genes significantly extends culture longevity and increases antibody titers by up to 2.1-fold (Marzluf J. et al., 2025).

Different G1 arrest strategies exhibit distinct trade-offs between stability, reversibility, cost, and productivity enhancement, with their relative performance systematically compared in Table 3. Genetic approaches such as MAT2A silencing enable stable long-term arrest but require prior cell line engineering. Chemical approaches represented by CDK4/6 inhibitors offer precise reversible control but incur higher process costs. Hypothermia is the most cost-effective option, though it delivers more modest productivity gains. For fed-batch cultures, hypothermia combined with sodium butyrate achieves an optimal balance between productivity and cell viability, while genetic induction is the preferred strategy for perfusion cultures.

**TABLE 3:** Cell cycle regulation and metabolic reprogramming strategies.

Regulation method	Mechanism of action	Metabolic effect	Production performance improvement
G1 arrest induction	CDK4/6 inhibitors, MAT2A silencing, Hypothermia, Sodium butyrate	Glycolysis downregulation, OXPHOS enhancement, reduced lactate	Increased qP, extended culture duration, Increased product titer
Apoptosis inhibition	BAX knockout, Bcl-2/Bcl-xL overexpression	Reduced programmed cell death, maintained mitochondrial function	Increased cell viability, extended production window
Autophagy regulation	Moderate autophagy activation, clearance of damaged organelles	Provision of emergency energy, maintenance of metabolic homeostasis	Enhanced cell viability under nutrient limitation
Cycle synchronization process	Dynamic intervention based on cell cycle distribution	Optimization of resource allocation, achievement of “growth-production” switch	Maximization of culture efficiency and product output

### 4.2 Metabolic signaling networks and cell cycle checkpoints exhibit bidirectional regulation

Cellular metabolic status actively feeds back to influence cell cycle progression through sophisticated sensing and signaling networks. The energy sensor AMP-activated protein kinase (AMPK) is activated under energy stress and can induce cell cycle arrest—typically in G1—by inhibiting pathways such as mTORC1, thereby curbing overall energy expenditure (Gwinn et al., 2008). Conversely, nutrient-sufficient conditions promote cell cycle progression via mTORC1 activation, driving anabolic processes (Villa et al., 2021). In large-scale cultures, cells frequently initiate apoptosis in response to stresses like nutrient limitation, metabolite accumulation, and hypoxia. This apoptotic response not only involves the mitochondrial pathway but is often preceded by metabolic-stress-induced activation of cell-cycle checkpoints (Avello et al., 2022). Engineering interventions at these integrated nodes therefore offer dual benefits. For instance, CRISPR-Cas9-mediated knockout of the pro-apoptotic gene BCL2-associated X (BAX) substantially delays cell death, extends culture duration, and increases target protein yield (Rahimi et al., 2023). Overexpression of anti-apoptotic proteins such as B-cell lymphoma 2 (Bcl-2) or B-cell lymphoma-extra large (Bcl-xL) stabilizes mitochondrial membrane potential and suppresses apoptosis (Du et al., 2017). Chromosomal site-specific integration strategies, which improve transgene stability and cellular stress tolerance (Huang et al., 2023), can also indirectly support healthier cell-cycle states by reducing metabolic burden and stress. Collectively, these approaches bolster cell survival under bioprocess stresses and help prevent premature metabolic collapse.

### 4.3 Synergizing cell cycle dynamics with process optimization

A clear understanding of cell-cycle–metabolism coupling directly informs culture-process optimization. Notably, cells in G1 phase generally exhibit higher metabolic activity than those in G0; thus, accurately distinguishing and regulating these two states can significantly enhance production outcomes (Avello et al., 2022). This observation suggests that maintaining cells in a metabolically active G1-arrested state may be more advantageous than allowing entry into quiescent G0. Studies on cell-cycle synchronization further reveal rhythmic oscillations in metabolic activity across cycle phases (Wang, 2022). By monitoring cell-cycle distributions, culture strategies can be dynamically adjusted—promoting proliferation when rapid biomass accumulation is desired, and synchronously inducing G1 arrest when high production is needed. Such temporal control over cellular metabolic resources maximizes overall process efficiency and product output.

### 4.4 Metabolic maintenance strategies during cell-cycle exit in high-density cultures

In the late stages of industrial high-density cultures, cells often exit the cell cycle, accompanied by a decline in metabolic function that limits both yield and product quality. These cells typically display mitochondrial dysfunction (Gopalakrishnan et al., 2024), impaired autophagy flux (Zhang et al., 2025), and weakened anabolism (Avello et al., 2022). Beyond inhibiting apoptosis, recent engineering efforts emphasize sustaining metabolic health after cycle exit. Moderately activating autophagy, for example, clears damaged organelles and supplies emergency energy, improving survival and metabolic function under nutrient-limited conditions (Junghans et al., 2019; Perez-Rodriguez et al., 2021). Overexpression of mitochondrial fusion proteins (e.g., MFN2) or key antioxidant enzymes preserves mitochondrial network integrity, alleviates oxidative stress, and helps maintain energy metabolism in post-mitotic cells (Son et al., 2015). Systematic tuning of both cell-cycle progression and programmed death pathways thus significantly enhances CHO cell stability and productivity in extended cultures (Elmore, 2007).

In summary, cell cycle regulation and metabolic reprogramming are deeply interconnected in CHO cells, as outlined in Table 3. Future work will increasingly employ systems biology tools such as multi-omics integration and synthetic biology strategies including reversible dynamic genetic circuits to precisely dissect and manipulate this complex interplay. Combining genome-scale metabolic models with cell cycle regulatory networks will help predict optimal engineering targets. By engineering the cell cycle to function as a metabolic master switch, it will be possible to achieve systematic and optimal resource allocation across the growth, arrest, and production phases, ultimately elevating CHO cell bioreactor performance to a new level (Moller et al., 2019).

## 5 Effect of cell culture conditions on CHO cell metabolism

The culture environment and process parameters exert a profound, multi-tiered regulatory influence on the metabolic network, growth kinetics, and the yield and critical quality attributes of recombinant proteins in CHO cells. These factors can be broadly classified as physicochemical conditions and nutrient supply. Through the rational design and dynamic control of these parameters—often augmented by metabolic engineering—cross-scale optimization can be achieved, from cellular metabolic foundations to overall bioreactor performance.

### 5.1 Regulatory role of physicochemical parameters

Key physicochemical parameters—pH, temperature, osmolality, and dissolved gas concentrations—directly shape metabolic flux and product quality by affecting enzyme kinetics, membrane transport, and signal transduction.

pH is critical for sustaining cellular metabolic homeostasis. Deviations from the typical optimum (pH 7.0–7.2) can reduce the activity of central metabolic enzymes, alter post-translational modifications, and disrupt glycosylation patterns. Specifically, pH changes alter the luminal pH gradient across the Golgi apparatus, which is essential for the sequential activity of glycosyltransferases and glycosidases. Even a 0.2 pH unit deviation can shift the balance between sialyltransferase and sialidase activity, leading to reduced terminal sialylation of recombinant proteins. Frequent or large pH fluctuations often inhibit growth, stimulate metabolic activity, elevate lactate production, and induce glycosylation variability, such as shifts in galactosylation (Jiang et al., 2018). For sialylation in particular, pH near 7.0 is generally optimal.

Temperature serves as a powerful lever for modulating cell-cycle progression and metabolic rates. Reducing temperature from 37 °C to 30 °C–33 °C (hypothermic culture) is a widely adopted industrial strategy to induce G1 arrest, lower overall metabolism, suppress apoptosis, and extend culture duration—thereby raising protein titer. However, lower temperatures can also dampen glycosyltransferase activity (Hossler et al., 2009), potentially compromising sialylation and necessitating careful balancing of yield and quality through process or genetic optimization.

Elevated osmolality (e.g., from added NaCl or concentrated media) imposes osmotic stress, generally inhibiting proliferation while increasing specific productivity (qP). Cells respond by synthesizing or importing compatible osmolytes (e.g., inositol, betaine), an energy-dependent process that can redirect metabolic flux. Hyperosmotic stress is frequently accompanied by increased ammonia accumulation, altered lactate metabolism, and a shift toward more basic glycosylation profiles (Alhuthali et al., 2021). Engineering overexpression of stress-responsive proteins such as HSP70 or antioxidant enzymes can bolster CHO cell tolerance to osmotic and other environmental challenges, enhancing process robustness (Kampinga et al., 2003).

Collectively, precise tuning of environmental parameters—combined with engineered stress resistance—supports efficient metabolism and consistent product quality by preserving enzyme activity, membrane integrity, and optimal flux distributions.

## 6 Byproduct accumulation and metabolic regulation

During the industrial-scale cultivation of CHO cells—particularly in high-density fed-batch and perfusion processes—the accumulation of metabolic byproducts presents a central challenge, limiting process scale-up, long-term cell viability, and recombinant protein quality consistency. These byproducts are not inert waste products; rather, they are dynamic outputs reflecting the state of the cellular metabolic network under stress or imbalance. Their formation and turnover provide insight into the efficiency of intracellular carbon, nitrogen, and energy flows. Systematic analysis of their generation mechanisms, elucidation of their multifaceted toxicities, and the development of prevention and mitigation strategies are therefore essential for optimizing CHO culture processes and achieving robust production. Lactate and ammonium represent the primary detrimental byproducts in CHO cultures; their accumulation inhibits growth and compromises product quality. A successful metabolic shift from lactate production to consumption is closely linked to culture longevity and final product yield.

### 6.1 Dynamic imbalance in lactate metabolism and its inhibitory effects

Lactate generation is a classic example of glycolytic “overflow metabolism.” Under nutrient-rich conditions, especially during rapid proliferation, CHO cells tend to generate ATP and biosynthetic precursors via high-rate glycolysis, yielding large amounts of pyruvate that are subsequently reduced to lactate by lactate dehydrogenase (LDH), concurrently regenerating NAD^+^ to sustain glycolytic flux. Although this metabolic phenotype supports rapid growth, it does so at the expense of metabolic efficiency (Marzluf J. et al., 2025).

The ideal metabolic trajectory involves a transition from net lactate production to net consumption in the mid-to-late culture phase, where cells re-oxidize lactate to pyruvate and subsequently oxidize it fully via the tricarboxylic acid (TCA) cycle. Frequently, however, this transition fails, resulting in persistent lactate accumulation that triggers multiple negative feedback loops. Literature-reported representative inhibitory concentration ranges for lactate in CHO batch and fed-batch cultures show that concentrations above 1.5–2 g/L typically begin to inhibit cell proliferation, while levels exceeding 3.5–4 g/L significantly reduce specific productivity and disrupt glycosylation patterns in CHO-K1 and CHO-S host cells (Marzluf J. et al., 2025). The exact toxic threshold depends on multiple factors including the specific CHO host cell line, medium formulation, feeding strategy, culture pH control strategy, and cultivation mode covering batch, fed-batch, and perfusion systems. For example, CHO-S cells generally exhibit higher lactate tolerance than CHO-K1 derivatives, and pH-controlled bioreactor cultures can withstand slightly higher lactate levels than uncontrolled shake-flask cultures.

The negative effects of excessive lactate accumulation are exerted through multiple coordinated mechanisms. (1) High lactate concentrations directly feedback-inhibit rate-limiting glycolytic enzymes such as hexokinase (HK) and phosphofructokinase (PFK), limiting glucose uptake and utilization and thereby constraining energy supply (Naik et al., 2023). (2) Lactate production is accompanied by substantial NADH generation. If mitochondrial oxidative capacity is inadequate or reducing-equivalent shuttles are inefficient, the cytosolic NADH/NAD^+^ ratio rises abnormally, inhibiting key NAD^+^-dependent enzymes such as glyceraldehyde-3-phosphate dehydrogenase (GAPDH) and stalling glycolysis. (3) Lactate accumulation directly lowers medium pH, and the base additions required for pH control elevate osmolality, imposing combined environmental stress. Moreover, elevated dissolved CO_2_ (pCO_2_), common in large-scale bioreactors, exacerbates intracellular acidification and has been shown to suppress mitochondrial electron-transport-chain function, fundamentally impeding lactate reoxidation (Cai et al., 2023).

In large-scale, high-density bioreactors, dissolved CO_2_ (pCO_2_) readily accumulates. High pCO_2_, exacerbated by excessive glucose and glutamine feeding that increases cellular respiration rates, reduces medium pH and causes intracellular acidification, impairing mitochondrial function and perturbing the NADH/NAD^+^ balance. This metabolic disruption impedes the shift from lactate production to consumption, sustaining lactate accumulation and limiting cell growth. Thus, online pCO_2_ monitoring and control are essential for maintaining metabolic stability in scaled-up processes.

### 6.2 Complex toxicity of ammonium ions and their effects on product quality

Ammonium ions arise primarily from deamination of amino acids such as glutamine, with glutaminase (GLS) playing a key role. In high-density, high-turnover culture systems, ammonium accumulation often outpaces cellular detoxification or assimilation capacity.

Representative toxic concentration ranges reported in literature indicate that extracellular ammonium concentrations above 1.5–2 mM begin to suppress cell growth, while levels exceeding 4–5 mM cause significant reductions in product sialylation and impair cell viability in CHO-K1 cells (Yang and Butler, 2000; Fan et al., 2015). Similar to lactate, the actual impact of ammonium is modulated by host cell line genotype, medium amino acid composition, feeding strategy, and Golgi luminal pH status. For example, GS-knockout host cells cultured in glutamine-free media generally have lower baseline ammonium production and thus higher tolerance to additional ammonium stress compared with wild-type cells.

Ammonium toxicity is complex and systemic, disrupting both the urea cycle and amino acid metabolism. Metabolically, high ammonium concentrations inhibit urea-cycle enzymes such as carbamoyl phosphate synthetase I (CPS I), perturbing nitrogen metabolism and potentially leading to amino-acid imbalances and accumulation of toxic intermediates like citrulline. At the organelle level, ammonium (NH_4_^+^) exists in equilibrium with free ammonia (NH_3_), which diffuses freely into cells and re-protonates, raising intracellular pH and interfering with enzyme activities and normal compartment function. Most critically for product quality, ammonium accumulation alters the luminal pH of secretory organelles such as the Golgi apparatus (McDermott and Butler, 1993; Zhang et al., 2021). Glycosyltransferases, especially sialyltransferases that add terminal sialic acids, are highly sensitive to local pH. Disruption of Golgi pH shifts these enzymes away from their optimal activity, directly reducing sialylation of recombinant proteins such as monoclonal antibodies and thereby affecting serum half-life and effector functions. Consequently, controlling ammonium accumulation is essential not only for maintaining cell health but also for preserving the critical quality attributes (CQAs) of biopharmaceuticals.

### 6.3 Targeted metabolic engineering and pathway remodeling strategies

Metabolic engineering offers precise solutions to reshape the metabolic network and reduce byproduct formation at its source. For lactate control, strategies focus on redirecting flux at the pyruvate node. Lactate dehydrogenase A (LDHA) expression can be downregulated via RNAi or CRISPR-Cas9 editing to directly curtail lactate synthesis. Alternatively, enhancing pyruvate flux into mitochondria—e.g., by overexpressing mitochondrial pyruvate carriers (MPC1/2) to improve transport (Bulte et al., 2020; Torres et al., 2025) or overexpressing pyruvate dehydrogenase (PDH) subunits to boost its activity—promotes oxidative metabolism. Overexpression of pyruvate carboxylase (PC) strengthens anaplerosis, supporting TCA-cycle flux and providing a route for lactate reentry. .Engineering of monocarboxylate transporters (MCTs) (the main membrane transporters for lactate) also represents a potential strategy to enhance lactate transport and metabolic homeostasis in high-density CHO cultures (Bulte et al., 2020).

For ammonium control, metabolic engineering aims to restructure nitrogen flux. A highly effective approach is the use of glutamine-free media coupled with metabolic adaptation (Ha et al., 2023), which forces cells to synthesize glutamine from glutamate and ammonium via the endogenous GS pathway, reducing net ammonium release and improving metabolic stability (Srila et al., 2023). This not only recycles ammonium intracellularly, drastically lowering its net release, but often improves metabolic stability and antioxidant capacity (Maralingannavar et al., 2019). Suppressing glutaminase (GLS) expression—via knockdown or CRISPR interference—further reduces ammonium generation at source (McDermott and Butler, 1993; Wu et al., 2018).

### 6.4 Synergistic intervention through process optimization and dynamic control

Glucose, as the primary carbon and energy source, strongly directs metabolic flux. High concentrations often trigger “overflow metabolism,” driving rapid glycolysis and excessive lactate formation. A restrictive feeding approach that maintains low glucose levels (e.g., 0.5–2 g/L) (Marzluf J. et al., 2025) can shift metabolism toward more efficient oxidative phosphorylation, reducing lactate accumulation and improving energy-yield efficiency. Balanced amino acid supply is equally crucial: amino acids act not only as protein-synthesis substrates but also as metabolic regulators. Recombinant protein production imposes high demands on specific amino acids (e.g., glutamine, asparagine, cysteine, tyrosine). Identifying rapidly depleted amino acids via metabolic flux analysis and designing dynamically balanced feeding media can prevent metabolic bottlenecks and toxic intermediate buildup, supporting sustained high productivity (Fan et al., 2015). For instance, adopting glutamine-free media forces reliance on the glutamine synthetase (GS) pathway, which curtails ammonia accumulation at the source and often upregulates adaptive metabolic networks, boosting antioxidant capacity and stabilizing glycosylation precursor pools (Taschwer et al., 2012).

Beyond macronutrients, precise supplementation of trace components and additives can directionally tune metabolism and product attributes. Manganese ions (Mn^2+^) are essential cofactors for many glycosyltransferases; their addition directly enhances Golgi-based glycan processing and is a common tactic to elevate antibody galactosylation and sialylation (Ehret et al., 2019). Adding galactose as a direct precursor can circumvent intracellular synthesis limits and promote glycan extension. Hormones or signaling modulators such as dexamethasone can indirectly modulate metabolic state and stress responses via glucocorticoid receptor signaling, improving product consistency (Kildegaard et al., 2016). Complex hydrolysates (e.g., cottonseed hydrolysate) deliver multi-component nutritional support; their peptides, lipids, trace elements, and undefined growth factors can holistically promote growth, extend culture lifespan, and strengthen stress resistance by affecting pathways like polyamine synthesis (Dhara et al., 2023).

Small-molecule metabolic modulators represent a sophisticated form of nutrient-based control. Histone deacetylase inhibitors (e.g., sodium butyrate) heighten transgene transcription by increasing chromatin accessibility, but they also induce cell-cycle arrest and broad metabolic reprogramming, requiring a trade-off between expression enhancement and growth suppression (Nmagu et al., 2021). Other modulators, such as 5′-deoxy-5′-(methylthio)adenosine (MTA), interfere with methylation cycles to induce cell-cycle arrest and selectively activate the pentose phosphate pathway. This markedly increases NADPH generation, supplying high-producer cells with ample reducing power for nucleotide/lipid synthesis and oxidative-stress defense, thereby raising specific productivity (Wijaya et al., 2021).

In summary, modern CHO medium optimization has evolved into a comprehensive metabolic engineering discipline. Through rational nutrient design and precise modulator addition, carbon, nitrogen, and energy flows are systematically directed toward product-synthesis pathways while concurrently refining quality attributes—forming the cornerstone of efficient, robust, and high-quality bioprocesses.

Beyond genetic modifications, intelligent process control provides a practical means of managing metabolic flux in industrial settings. Excessive feeding is a major cause of byproduct accumulation in industrial fed-batch processes, and is tightly linked to several key bioprocess parameters. Glucose feed rate is a primary influencing factor, as overly high glucose feeding triggers the Crabtree effect, where cells maintain high glycolytic flux even under sufficient oxygen supply, leading to massive lactate overflow. Glutamine feed level also plays a critical role, since excessive glutamine supplementation accelerates glutaminolysis, resulting in rapid ammonia accumulation and subsequent Golgi pH disruption. Feed osmolality is another non-negligible parameter, as over-concentrated feed media increases culture osmolality, inducing cellular metabolic stress that further exacerbates lactate and ammonia production. Feeding timing also matters greatly, and misaligned feeding—such as maintaining high feed rates after cells enter stationary phase and reduce metabolic demand—leads to nutrient buildup and waste accumulation.

Central to solving these issues is dynamic feeding, where nutrient addition is adjusted in real time based on online or at-line measurements of glucose, lactate, and ammonium. Raman spectroscopy, as a non-invasive online detection technology, has been used to establish universal quantitative models for key metabolites in GS-KO CHO fed-batch platforms, enabling accurate real-time feedback to guide dynamic feeding control (Webster et al., 2018). The goal is to maintain substrate concentrations within a low, non-limiting “optimal window,” thereby avoiding nutrient-driven overflow metabolism and steering cells toward more efficient metabolic states (Young, 2013). For example, keeping glucose at low levels (e.g., 0.5–2 g/L) encourages greater reliance on oxidative phosphorylation.

Several representative industrial applications of dynamic feeding strategies have demonstrated significant benefits for cell performance and product quality. Raman spectroscopy-based real-time feedback feeding, applied to GS-knockout CHO cell lines producing monoclonal antibodies, maintains glucose concentration within a narrow optimal window of 1.0–2.0 g/L throughout fed-batch culture. Compared with conventional bolus feeding, it reduces peak lactate accumulation by 40%, increases final antibody titer by 25%, and reduces batch-to-batch variation in core fucosylation level by over 30% in GS-knockout CHO cell lines (Webster et al., 2018; Domjan et al., 2022). Viable cell density-aligned dynamic feeding, which is widely adopted in industrial biomanufacturing, adjusts feed rates proportionally to real-time viable cell density to match cellular metabolic demand. In bispecific antibody production using CHO-S cells, it reduces peak ammonium concentration by 35% and decreases product aggregate levels by 20% compared with fixed-rate feeding. As for alternative carbon source feeding, partial replacement of glucose with galactose moderates glycolytic overflow and promotes lactate consumption. In CHO-K1 cell fed-batch cultures, this strategy reduces peak lactate levels by 50% and improves terminal sialylation of recombinant glycoproteins by 15%–20% (Altamirano et al., 2006). Similarly, using glutamate or stabilized dipeptides such as alanylglutamine in place of glutamine enables slower, more controlled amino acid release, preventing the rapid degradation and ammonium spikes associated with free glutamine.

### 6.5 Auxiliary application of small-molecule inhibitors and chemical modulators

Small-molecule compounds serve as rapid-response tools for fine-tuning metabolic phenotypes. The hexokinase inhibitor 2-deoxy-D-glucose (2-DG) reversibly reduces glycolytic flux and lactate generation; its pulsed addition during high-lactate phases shows promising application potential. Dichloroacetate (DCA), an inhibitor of pyruvate dehydrogenase kinase (PDK), activates the PDH complex, shifting carbon flux toward the TCA cycle (Buchsteiner et al., 2018). For ammonium control, glutaminase inhibitors such as 6-diazo-5-oxo-L-norleucine (DON) can be employed (Cederkvist et al., 2022), though their broader metabolic impacts require careful evaluation.

Looking forward, byproduct management will evolve from reactive measures toward predictive-preventive systemic engineering. This shift will depend on deeply integrating multi-omics analyses, genome-scale metabolic models (GEMs), and process analytical technologies (PAT). Linking model predictions with real-time sensor data will enable adaptive control systems that pre-empt metabolic drift and auto-adjust process parameters, paving the way for intelligent, robust production. The ultimate aim is to engineer CHO cells into “green” cell factories—with balanced carbon-nitrogen metabolism, strong self-regulation, and minimal byproduct formation—thereby delivering higher productivity, superior product quality, and more stable manufacturing processes to the biopharmaceutical industry (Table 4).

**TABLE 4:** Byproduct accumulation and control strategies in CHO cell metabolic engineering.

Byproduct/Process stressor	Main source	Negative impact	Control strategies
Lactate	Glycolytic overflow, high LDH activity	Inhibits glycolytic enzymes, acidifies medium,	LDH knockdown, PDH/PC overexpression, low-glucose feeding
Ammonium	Glutamine deamination, amino acid catabolism	Alters Golgi pH, inhibits glycosylation	Glutamine-free media, GS pathway enhancement, GLS inhibition
CO2	Oxidative metabolism, large-scale accumulation	Intracellular acidification, inhibits mitochondrial	Online pCO2 control, optimized aeration
Osmolality	Base addition, concentrated feeding	Inhibits proliferation, alters glycosylation	Optimized feeding, low-salt media
Reactive oxygen species (ROS)	Mitochondrial respiration, ER stress	Damages proteins, induces apoptosis	Antioxidant overexpression, ROS scavengers
Host cell proteins (HCPs)	Cell lysis, active secretion	Co-purification, immunogenicity	HCP-minimized chassis, optimized harvest

## 7 Application of emerging systems biology technologies in CHO cell metabolic engineering

Advances in synthetic biology, systems biology, and data science are fundamentally reshaping CHO cell metabolic engineering. The traditional trial-and-error model, focused on single targets, is giving way to systematic approaches grounded in high-throughput data, computational modeling, and rational design. These emerging technologies not only improve the efficiency and precision of cell engineering but also allow a global perspective on metabolic networks, enabling the design of next-generation cell factories with superior performance.

Integrated high-throughput multi-omics analysis is central to probing metabolic network states. By systematically profiling CHO cells at genomic, transcriptomic, proteomic, and metabolomic levels, researchers can identify key metabolic features and regulatory nodes associated with high-yield, stable-production phenotypes across the genome. For instance, comparing metabolomic and transcriptomic profiles of high- and low-producer clones reveals patterns such as enhanced central-carbon flux, improved oxidative phosphorylation, and reprogrammed amino-acid metabolism in high-yield lines. Time-series multi-omics further captures dynamic adaptations of the metabolic network during culture, providing direct evidence for stage-specific bottlenecks (Huang et al., 2017; Gopalakrishnan et al., 2024). Tools like pathway-enrichment analysis and weighted gene co-expression networks help extract regulatory hubs from large datasets, offering high-value targets for engineering interventions (Vandereyken et al., 2023).

Genome-scale metabolic models (GEMs) and their extensions are now indispensable computational platforms. Foundational CHO GEMs were built on the first draft CHO-K1 genome sequence and the Chinese hamster reference genome (Xu et al., 2011; Lewis et al., 2013), complemented by the first public CHO cell line transcript database constructed by Rupp et al. (Rupp et al., 2014), which integrated transcriptomic data from multiple cell lines and culture conditions to significantly improve genome annotation accuracy and gene boundary definition. The consensus model iCHO2291 (Hefzi et al., 2016) subsequently became the community standard (Strain et al., 2023). On this basis, dynamic constraint-based modelling frameworks have been further developed to simulate time-course metabolic changes during fed-batch culture, and can accurately predict growth dynamics, optimal medium composition and intracellular flux distributions across different CHO cell clones (Yasemi and Jolicoeur, 2023). Using methods such as flux-balance analysis, they simulate maximum theoretical growth or product yield under defined nutrient conditions and predict gene-knockout or overexpression strategies to meet specific production goals, e.g., maximizing antibody synthesis flux. More importantly, integrating metabolic models with transcriptomic data (e.g., through enzyme-constrained models) or coupling them with models of glycosylation, secretion, and other processes enables end-to-end prediction from substrate uptake to final product formation. Such integrated models can virtually screen thousands of genetic and medium combinations, drastically narrowing experimental space and accelerating rational design.

The field achieved a major breakthrough in 2026 with the release of iCHO3K, the most comprehensive CHO GEM to date (Di Giusto et al., 2026). This model contains 11,004 reactions, 7,377 metabolites, and 3,597 genes, and uniquely integrates 3,489 experimentally determined protein 3D structures to enable allosteric regulation predictions. When applied to the lactate-free CHO-ZeLa cell line, iCHO3K revealed reduced glycolytic flux, enhanced TCA cycle activity, and elevated NADH/phosphoenolpyruvate (PEP) ratios. Critically, it identified a previously unknown allosteric PEP binding site on phosphofructokinase (PFK), directly linking the lactate feedback inhibition mechanism (Section 6.1) to the pyruvate node regulation (Section 2.1). This discovery explained why PFK overexpression often fails to improve glycolytic efficiency in high-lactate cultures and provided a novel target for rational metabolic engineering.

Real-time and dynamic metabolic monitoring technologies are shifting metabolic control from “static optimization” to “dynamic adaptation.” Fluorescent-protein-based biosensors track key metabolites (e.g., ATP, NADH, glucose) in living cells, while online mass spectrometry and Raman spectroscopy enable near-minute-level monitoring of dozens of extracellular metabolites (Domjan et al., 2022). These real-time data streams support closed-loop feedback systems that automatically adjust feeding rates or induction timing based on the cell’s metabolic state—for example, responding to early lactate-accumulation signals—keeping metabolism within an optimal high-yield, low-byproduct range and markedly improving process robustness and product consistency (Domjan et al., 2020).

The expanding CRISPR toolbox offers unprecedented flexibility for precise metabolic network reconstruction. Beyond knockout, CRISPR activation/interference systems allow fine-tuning of endogenous gene expression (Lee et al., 2015). Base editing and prime editing achieve precise point mutations without DNA double-strand breaks, enabling subtle adjustment of metabolic-enzyme activity or substrate specificity (Kantor et al., 2020). Coupled with single-cell sequencing and high-throughput screening, these tools support genome-wide functional screens that systematically map gene-phenotype relationships and uncover novel engineering targets (Rees and Liu, 2018).

Deep integration of artificial intelligence and machine learning is catalyzing a new, data-driven paradigm for cell engineering. Algorithms such as deep learning extract complex nonlinear relationships from historical multi-omics data, process parameters, and final yield/quality metrics to build high-precision predictive models. These models not only forecast outcomes of specific engineering strategies but also enable inverse design: given desired product attributes, they recommend optimal gene-editing combinations and culture schemes. This shifts the metabolic-engineering cycle from “analyze-design-build-test” toward “intelligent design-automated construction-predictive validation.”

### 7.1 High-throughput pooled CRISPR screening for target discovery

Traditional candidate-gene approaches are limited by prior knowledge, whereas pooled CRISPR screens eliminate clonal noise and enable unbiased mapping of gene-phenotype relationships. Marzluf et al. developed a stable Cas9 pool workflow that compresses screening timelines from 9 to 5 weeks and increases throughput ∼2.5-fold. This platform was validated by reproducing the benefits of fibronectin 1 (FN1) knockout (extended culture duration and higher titers) without single-cell cloning variability (Marzluf J. P. et al., 2025).

In this landmark genome-wide screen of 17,761 expressed genes in both shake-flask and 3 L perfusion cultures, Marzluf et al. identified 235 growth-enhancing knockouts and defined the CHO_EG2025 benchmark set of 3,110 essential fitness genes (Marzluf J. et al., 2025). This work provides the first unbiased atlas of CHO cell fitness genes and represents a paradigm shift from targeted to systems-level target discovery.

When combined with automated microscale bioreactor and optofluidic screening platforms, pooled CRISPR workflows enable end-to-end high-throughput target validation and cell line generation in less than 8 weeks.

### 7.2 Host cell protein (HCP) minimization and clean chassis engineering

HCPs are a major challenge in biopharmaceutical manufacturing, as they can co-purify with the target product and trigger immunogenic responses. Marzluf et al. used quantitative proteomics to identify 62 abundant, persistent HCPs, and validated 36 as compatible with normal growth when knocked out. Multiplex editing of up to 11 genes simultaneously achieved a 57%–85% reduction in released HCPs without compromising fed-batch performance. Notably, FN1 knockout was doubly beneficial, reducing HCP levels while extending culture duration (Marzluf et al., 2026).

### 7.3 Artificial intelligence and machine learning applications

AI/ML models now provide concrete, actionable insights for CHO engineering. Li et al. developed a deep learning model that predicts antibody glycosylation profiles with >90% accuracy using transcriptomic and process data (Li et al., 2024). Richelle et al. (2026) created a kinetic-ML hybrid model that forecasts lactate and ammonia accumulation 72 h in advance with R^2^ > 0.92, enabling proactive feeding adjustments that reduced byproduct levels by 35% in industrial fed-batch cultures (Richelle et al., 2026). Kotidis integrated central metabolism and glycosylation models to predict how genetic modifications affect both productivity and product quality (Kotidis et al., 2023).

In summary, the convergence of systems biology, synthetic biology, and artificial intelligence is elevating CHO cell metabolic engineering to a new level. A closed-loop workflow—spanning panoramic measurement, model simulation, dynamic sensing, intelligent decision-making, and precise manipulation—provides the capacity to understand and design cell factories with unprecedented depth (Table 5). The future lies in further dissolving barriers between technologies and data types to construct a “digital twin” of the cell, from gene sequence to industrial batch. This will ultimately enable customized, predictable optimization of CHO cell production performance, laying the groundwork for intelligent biopharmaceutical manufacturing.

**TABLE 5:** Future trends and challenges in CHO cell metabolic engineering.

Direction	Development trends	Challenges
Systems metabolic engineering	Multi-omics integration AI prediction Dynamic regulation	High complexity of metabolic networks, difficulty predicting multi-target synergy, need for improved model reliability
Smart cell factories	Metabolite sensors Closed-loop control Digital twin systems	High cost of real-time monitoring technology, insufficient stability of regulatory circuits
Personalized metabolic Design	Customizing metabolic networks for product attributes Process matching optimization	Cell line stability, scale-up effects, regulatory and compliance requirements
Green and sustainable production	Byproduct minimization Efficient C/N utilization Serum-free media	Metabolic load balancing, cell adaptability, cost control

## 8 Summary and prospects

CHO cell metabolic engineering has evolved from a discipline focused on localized, single-target interventions into a comprehensive framework for the systemic design and global optimization of cell factories—a core enabler of modern biopharmaceutical manufacturing. This review has examined five key dimensions that shape the CHO metabolic network: (1) engineering central metabolism to optimize energy and redox cofactor supply; (2) glycosylation engineering to precisely control glycan synthesis and ensure critical product quality attributes; (3) cell-cycle regulation to drive the strategic shift from proliferation to production; (4) rational culture condition design to fine-tune metabolic flux and enzyme activity; and (5) proactive byproduct management to sustain metabolic homeostasis during prolonged cultivation. These dimensions are interconnected and mutually reinforcing, together forming a multi-level engineering system that enhances CHO cell production performance.

Despite these significant advances, long-term stability of recombinant CHO cell lines remains one of the most pressing unresolved challenges in industrial biomanufacturing. Persistent challenges in transcription factor-based metabolic engineering specifically include unpredictable off-target effects of multi-transcription factor engineering, difficulty in precise dynamic regulation of TF activity across different culture phases, and poor scalability of lab-validated TF modification strategies to large-scale industrial bioreactors. Over extended culture periods (typically >50 passages), engineered lines frequently exhibit declining productivity, altered glycosylation profiles, and increased batch-to-batch variability. This instability arises from two primary mechanisms: genetic mutations and epigenetic silencing.

Epigenetic silencing, in particular, is responsible for the majority of transgene expression loss in industrial cell lines. DNA methylation of promoter regions and histone deacetylation can gradually repress transgene transcription, even in clones with high initial expression levels. Veith demonstrated that 68% of unstable CHO clones showed significant hypermethylation of the CMV promoter within 30 passages. Additionally, metabolic drift—gradual changes in cellular metabolism due to adaptive evolution—can alter nutrient utilization and byproduct formation, leading to inconsistent product quality (Veith et al., 2016).

Several strategies have been developed to mitigate these issues. Site-specific integration into transcriptionally active safe harbor loci reduces the risk of position-effect silencing by avoiding heterochromatic regions. The use of insulator elements flanking the transgene expression cassette can also block the spread of repressive chromatin marks. Epigenetic engineering approaches, such as knockout of the histone deacetylase Bcor or overexpression of DNA demethylases, have been shown to significantly improve long-term transgene expression stability without compromising productivity (Veith et al., 2016; Kim et al., 2024).

Looking forward, the integration of emerging technologies will propel metabolic engineering toward greater intelligence and precision. The deep fusion of artificial intelligence with multiscale modeling (Fouladiha et al., 2021) will enable high-fidelity predictive models built from integrated multi-omics data (Rejc et al., 2017), shifting the paradigm from empirical trial-and-error to rational prediction and allowing virtual screening of optimal targets and personalized metabolic designs. Synthetic biology tools including reversible dynamic genetic circuits and advanced CRISPR systems such as base editing and prime editing will support spatiotemporally specific, reversible finetuning of metabolic pathways, enabling adaptive optimization of cellular metabolic states (Yeo et al., 2020). Furthermore, systems biology-guided personalized metabolic optimization will combine genome-scale metabolic models with real-time process analytics to achieve optimal matching of cell factory, process, and product, particularly for tailored attributes such as custom glycoforms, thus bridging the design path from genotype to industrial phenotype (Weston and Thiele, 2023).

Through the integrated application of these interdisciplinary technologies, next-generation CHO platforms can be envisioned: cell factories that intelligently direct metabolic flux toward product synthesis, offer highly controllable quality attributes, and demonstrate exceptional robustness throughout production. Ultimately, such efficient, intelligent, and sustainable cell factories will provide decisive support for the intelligent manufacturing and innovative development of biopharmaceuticals.
